# Impact of Parental Primary Spoken Language on Postoperative Pain Management in Children, a Retrospective Cohort Study

**DOI:** 10.3390/children9050739

**Published:** 2022-05-18

**Authors:** Robert E. Shaw, Steven J. Staffa, Viviane G. Nasr, Cynthia Tung

**Affiliations:** 1 Division of Pediatric Anesthesia, Department of Anesthesiology, University of Wisconsin, Madison, WI 53792, USA; 2 Department of Anesthesiology, Critical Care and Pain Medicine, Boston Children’s Hospital, Boston, MA 02115, USA; steven.staffa@childrens.harvard.edu (S.J.S.); viviane.nasr@childrens.harvard.edu (V.G.N.); cynthia.tung@childrens.harvard.edu (C.T.)

**Keywords:** anesthesia, acute pain, surgery, disparities

## Abstract

Background: Healthcare providers encounter varying languages every day in patient care. The goal of this study was to examine whether a difference exists in pain scoring and treatment amongst pediatric patients whose families’ primary language was not English. We hypothesized that patients of both Arabic-speaking (AS) and Spanish-speaking (SS) backgrounds received higher pain scores and higher daily opioid equivalents (OEs) postoperatively compared to English-speaking (ES) patients. Methods: This was a retrospective cohort study of patients undergoing surgery for treatment of various esophageal and airway disorders from 2014 to 2019. Records were queried for patients undergoing thoracotomies for treatment of esophageal and airway disorders. Analysis was focused on the three most common languages in this selected population: English, Arabic, and Spanish. Propensity score matching was utilized for comparisons of pain scores between AS, SS, and ES patient groups. Primary outcomes were opioid equivalents (OEs) and pain scores postoperatively. Multivariable median regression analysis was used to perform an adjusted comparison of pain scores as well as OEs. Results: A total of 610 patient encounters were included in our analysis. In propensity matched analysis, there were no significant differences in OEs between all groups postoperatively. However, statistically significant higher pain scores were reported in the ES group matched AS groups. Conclusions: Language differences did not lead to difference in pain assessment and treatment.

## 1. Introduction

The United States has become a destination for international patients needing world class medical care. With the arrival of different nationalities, cultures, and races, differences in languages spoken by patients and their families is expected. A large quaternary children’s hospital in New England received 2700 international patients from over 110 countries in 2019 [1]. In addition to international patients, the U.S. has more than 350 different spoken languages [2]. In Massachusetts, just under 10 percent of the adult population lacks proficiency in English, amounting to over 500,000 people [3,4]. With this multitude of languages spoken, healthcare providers need to find adequate ways to communicate with their patients. Many resources are available, including on-site interpreters, video and audio interpreting services, a patient’s family members, friends, or other hospital staff. However, these resources are not continuously available at the bedside and are mainly utilized during procedural informed consents or treatment planning.

While racial and ethnic disparities in pain management have been well-documented [5,6,7,8,9,10,11,12,13,14,15,16,17,18], there is a paucity of data on these disparities in pediatric perioperative pain control [13,14]. Furthermore, pediatric studies assessing the impact of primary spoken language on perioperative pain management are limited [15,16,17]. Proficiency in English by a patient’s parent seems to be a factor in access to healthcare, quality of healthcare, and parental satisfaction of their child’s care [15]. Interviews conducted with Latina mothers in an urban setting also showed parents felt more satisfied with primary care experiences than specialty or emergency care, partly because of challenges with unfamiliarity in navigating such specialty services [15].

Many families with different primary languages travel to have a wide array of surgical procedures performed on their children at our quaternary children’s hospital. Arabic-speaking (AS) and Spanish-speaking (SS) patients come for treatment of esophageal and airway disorders, ranging from primary tracheoesophageal fistula (TEF) ligations and esophageal atresia repairs, to more complex, multi-staged procedures for management of long gap esophageal atresia. However, language may be a barrier in communication and hence, pain management.

While previously published studies show racial or ethnic disparity in pain recognition and treatment [19,20], race and ethnicity are not interchangeable with primary spoken language. Based on our institutional and clinical experiences with patients of diverse cultural backgrounds, we have noted potential over-scoring, and potential over-treatment of pain in non-English speakers, aiming to ensure adequate analgesia and patient satisfaction. This is in line with other institutional experiences where patients who are less proficient in English had higher chance of receiving pain treatment than those who were more proficient in English [15].

Recognizing cultural differences and potential language barriers when providing patient care, we sought to observe whether there were differences in the way pain is recognized and treated for non-English-speaking patients and their families compared to their English-speaking (ES) counterparts. We hypothesized that patients of both AS and SS families received higher pain scores and higher daily opioid equivalents (OEs) over the course of the first 48 h postoperatively, following TEF repair.

## 2. Materials and Methods

Following Institutional Review Board approval, this retrospective cohort study was a review of all patients who underwent surgery for open repair of TEF and/or open repair of esophageal atresia between 2014–2019. The institutional surgical approach to TEF and esophageal atresia is consistent, via open thoracotomy, and therefore, a consistent pain profile is expected. In addition to a consistent surgical technique, a select team of surgeons and anesthesiologists performed these operations during this time period, providing consistent surgical skills and anesthetic management among all patients. Regional anesthesia adjuncts used were either an epidural or paravertebral catheters, at the discretion of the anesthesiologist of record, with continuous local anesthetic infusion for at least 48 h. The electronic medical record was queried for patient encounters of those who underwent open thoracotomy for the following procedures (Current Procedural Terminology (CPT) codes in parenthesis): cervical tracheopexy (31750), thoracic tracheopexy (31760), aortopexy (33800), repair esophageal atresia and TEF, congenital (43314), repair esophageal atresia without TEF (43313), TEF repair, cervical approach (43305), and TEF repair, thoracic approach, not congenital (43312). Patients of all age groups and all primary spoken languages were included in data collection. Subsequently, analysis was then focused on the three most common languages in this selected population: English, Arabic, and Spanish. The primary variable of interest was parental primary language. As these patients tend to have multiple procedures over different encounters, only the initial and secondary encounters were included. Patient encounters were excluded from data analysis if they involved simultaneous cardiac surgery or if the surgical approach was a sternotomy. Data collection included age, weight, sex, primary language spoken, and concomitant regional anesthesia at time of surgery, surgical duration, postoperative ventilation method, and postoperative destination (intensive care unit versus inpatient floor). Additionally, the presence or absence of regional anesthesia at the time of surgery, as well as any diagnosis of chronic pain and developmental delay diagnosed after 1 month of age, were queried using International Classification of Disease (ICD) 9 and 10 codes (Appendix A). Primary outcomes were opioid equivalents/kilogram including both bolus and infusions. Secondary outcomes were pain scores assessed using the Face, Legs, Activity, Cry, and Consolability (FLACC) Score, Numerical Rating Scale (NRS), and Wong–Baker FACES scale [21,22,23] at 6, 12, 24, 36, and 48 h postoperatively. Depending on the scoring system used, there was a mix of observational pain scoring (FLACC and FACES scale) and verbal reporting of pain (NRS scale).

### Statistical Analysis

Continuous data within each group were presented as medians and interquartile ranges, and categorical data were presented as frequencies and percentages. Univariate comparisons were done using the Wilcoxon rank sum test, Fisher’s exact test, or the Chi-square test, as appropriate. Propensity score matching was utilized for comparisons of pain scores between AS, SS, and ES patient groups. Propensity score matching (PSM) was performed using 1:5 between the AS and ES patient groups, and between the SS and ES patient groups with nearest neighbor matching and using the following matching variables: age, weight, sex, procedure type, encounter number, developmental delay, and history of chronic pain. Standardized mean differences (SMD) were calculated before and after propensity score matching, with values less than 0.2 considered as a reflection of good balance between the matched groups. Multivariable median regression analysis was used to perform an adjusted comparison of pain scores as well as OEs between AS, SS, and ES patient groups while accounting for clustering of multiple encounters per patient. A sensitivity analysis was performed using traditional multivariable median regression modeling, each adjusting for the following confounding variables: age, weight, sex, procedure, encounter number, developmental delay, history of chronic pain, regional use, opioid infusions, surgical duration, ventilation, and ICU admission.

Results from median regression analyses were presented as adjusted difference in medians between comparison groups with corresponding 95% confidence intervals and *p* values. A two-tailed *p* < 0.05 was considered statistically significant. Stata (version 16.0, StataCorp LLC., College Station, TX, USA) was implemented for all statistical analyses.

## 3. Results

**Figure 1:**
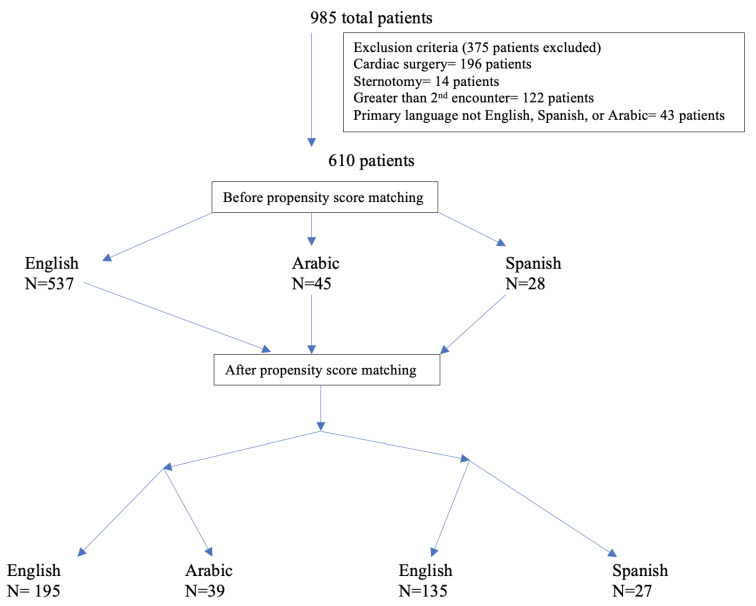
Number of patients with exclusion criteria and propensity matching applied.

A total of 985 patient encounters were queried during data collection. After exclusion criteria were applied, a total of 610 total patient encounters (537 ES patients, 45 AS patients, and 28 SS patients) were utilized during data analysis (Figure 1). Of the 610 encounters analyzed, 210 (39%) were female and 400 (61%) were male. The mean age was 0.9 years. The most common spoken language spoken was English and 10% of encounters were from non-English-speaking backgrounds.

Median OEs per kilogram of body weight and median pain scores for each language group are reported in Table 1. Of note, out of the total reported 13,574 pain scores, 11,899 (87.6%) were FLACC scores. Other scores utilized were NRS (982 pain scores, 7% of total scores) and Wong–Baker Generalized pain scores (534 pain scores, 4% of total score). Opioid equivalents were both presented as continuous opioid infusions (morphine, fentanyl or hydromorphone) and as needed bolus opioids. A total of 105 of 961 had only continuous opioid infusions, 255 encounters had only bolus opioids, and 554 encounters had both bolus and continuous opioids. There was minimal difference in opioid consumption and lower pain scores in the AS patients compared to ES patients. There was a higher opioid consumption by SS patients compared to ES patients at all measured time periods in the first 48 h postoperatively, but no significant difference in pain scores between SS and ES patients.

**Figure 2:**
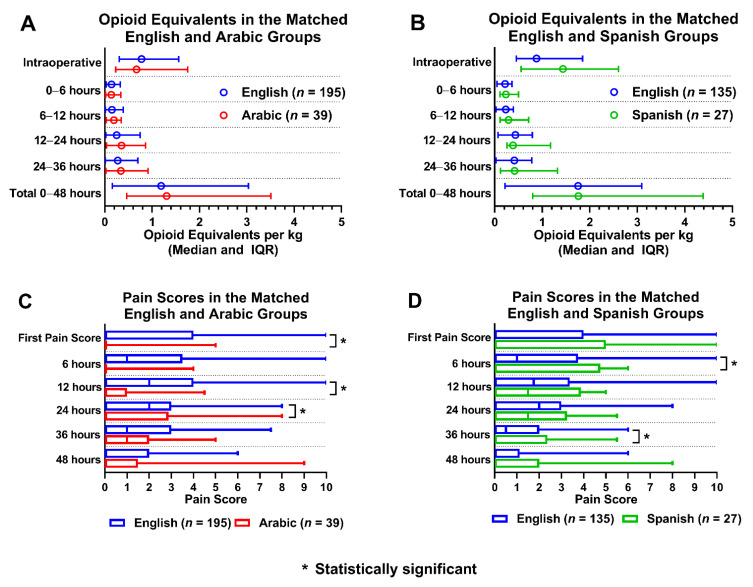
Opioid equivalents and pain score comparisons amongst propensity matched language groups. (**A**): Opioid equivalent per kg vs. time in matched English and Arabic groups; (**B**): Opioid equivalent per kg vs. time in matched English and Spanish groups; (**C**): Pain score vs. time in matched English and Arabic groups; (**D**): Pain score vs time in matched English and Spanish groups.

Baseline variables of the patients before and after propensity score matching (PSM) are presented in Table 2 and Table 3, and reflect a balance of all variables between the matched groups. A total of 195 ES patients were matched to 39 AS patients (5 ES patients to 1 AS patient), and a total of 135 ES patients were matched to 27 SS patients (5 ES patients to 1 SS patient). There was minimal difference in OEs between the AS and ES patients, and no significant difference in OEs between SS and ES patients (Figure 2). Pain scores were lower for AS patients when compared to ES patients, statistically significant for the first pain score, 12 h and 24 h postoperatively. Finally, pain scores were higher in SS patients compared to ES patients, and statistically significant for 6 and 36 h postoperatively for SS patients (Figure 2).

**Figure 3:**
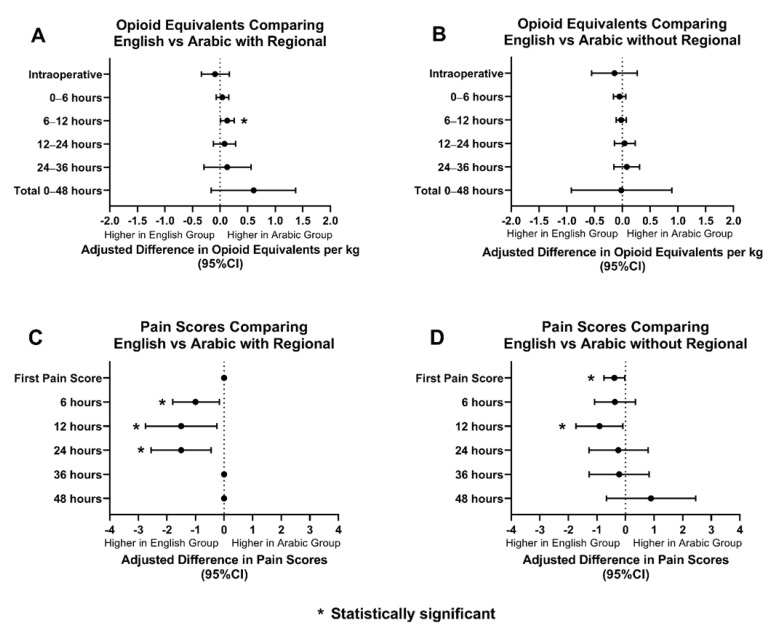
Opioid equivalents and pain score comparisons amongst ES and AS patients with and without regional anesthesia. (**A**): Adjusted difference in opioid equivalents per kg vs. time in English vs. Arabic patients with regional anesthesia; (**B**): Adjusted difference in opioid equivalents per kg vs. time in English vs Arabic patients without regional anesthesia; (**C**): Adjusted difference in pain score vs. time in English vs. Arabic patients with regional anesthesia; (**D**): Adjusted difference pain score vs. time in English vs. Arabic patients without regional anesthesia.

**Figure 4:**
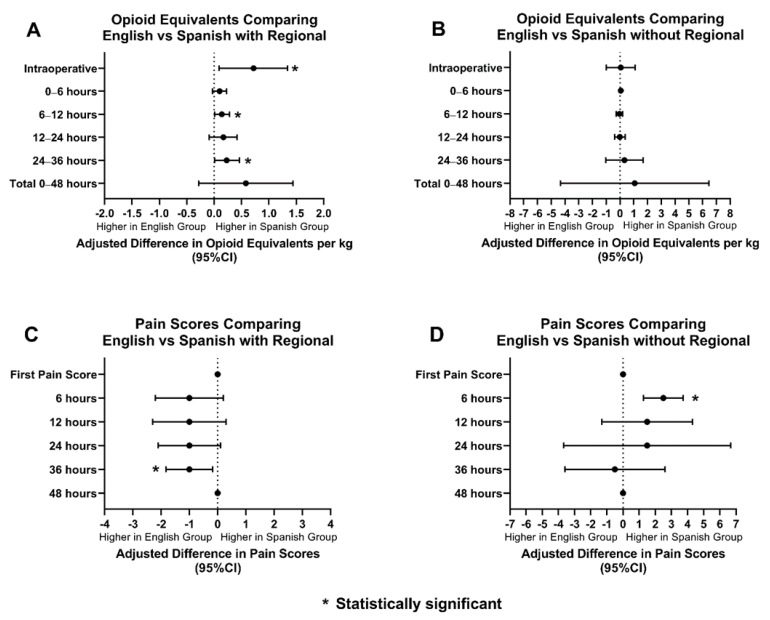
Opioid equivalents and pain score comparisons amongst ES and SS patients with and without regional anesthesia. (**A**): Adjusted difference in opioid equivalents per kg vs. time in English vs. Spanish patients with regional anesthesia; (**B**): Adjusted difference in opioid equivalents per kg vs. time in English vs. Spanish patients without regional anesthesia; (**C**): Adjusted difference in pain score vs. time in English vs. Spanish patients with regional anesthesia; (**D**): Adjusted difference pain score vs. time in English vs. Spanish patients without regional anesthesia.

Regional anesthesia was utilized as an adjunct in 68% of ES patients (369/537), 57% of AS patients (26/45), and 75% of SS patients (21/28). When the groups with regional anesthesia were compared, OEs between AS and ES patients were similar amongst the two groups. AS patients had statistically significant lower pain scores than ES patients for the first 24 h postoperatively (Figure 3). SS patients had higher OEs intraoperatively, at 12 h, and at 36 h postoperatively. SS patients had statistically significant lower pain scores only at 36 h postoperatively compared to ES patients. (Figure 4).

OEs and pain scores between AS and ES patients, without regional anesthesia, were also compared. AS patients received similar OEs when compared to ES patients. AS patients received statistically significant lower scores for the first pain score and at 12 h postoperatively (Figure 3). SS patients and ES patients had comparable OEs and pain scores, however, SS patients did have higher pain scores at 6 h postoperatively (Figure 4).

To further address variables that may affect pain scores and OEs, a sensitivity test using multivariable regression analysis was performed, adjusting for age, weight, sex, developmental delay diagnosis, chronic pain diagnosis, surgical duration, procedure type, first or second encounter, regional anesthesia utilization, ICU admission, postoperative ventilator status, and postoperative continuous opioid infusion (Table 4). OEs and pain scores between AS and ES, as well as SS and ES were comparable. The only statistically significant differences were at 12 and 24 h, when AS patients reported lower pain scores than ES patients, which mirrored the same findings in the PSM analysis.

## 4. Discussion

AS and SS patients had comparable pain scores and similar OEs per kilogram as their ES counterparts after surgical repair for conditions including and related to esophageal atresia (EA)/tracheoesophageal fistula (TEF). While unmatched data showed overall higher reported pain scores among ES patients compared to AS patients, there was no difference in pain scores or OEs between these two groups after propensity score matching. Although the additional multivariate analysis showed statistically significant higher pain scores for ES patients at 12 and 24 h postoperatively, there was no clinical significance in such differences. The interquartile range of pain scores between AS–ES and SS–ES compared groups ranged from 0 to 4 and 0 to 5, respectively. Considering a pain score of 0–4 is considered mild, large doses of opioids would not be administered within this range, and our results reflected this. Although a pain score of 5 is considered moderate pain, the few SS patients did not contribute to a significant increase to overall requirement of opioids. Only four SS patients had a first pain score of 5: one had a 6 h postoperative pain score of 5 and one had a pain score of 5 at both first pain score and 6 h postoperatively. Similarly, unmatched data showed SS patients had higher OEs, and initial analysis revealed no difference in pain scores or OEs between SS and ES patients, except for higher pain scores among ES patients at 6 and 36 h postoperatively (IQR 0–5, *p* = 0.029 and 0–2, *p* = 0.016, respectively). After multivariate analysis, there was no statistical difference in pain scores between the two groups, which supported that this difference, again, may not be clinically significant.

Despite the difference in languages in this surgical patient population, the lack of clinical difference in pain scores and management may be due to the consistency in surgical technique and anesthetic management. Patients with esophageal atresia and TEF were consistently operated on and cared for by the same small group of surgeons and anesthesiologists, as part of the esophageal atresia team. Thus, there was little variation in surgical technique, anesthetic plan and management, and postoperative management including opioid order sets. In this study, standardization of surgical and anesthetic management may have played a role in preventing differences in pain management among ES and non-ES patients.

There are several limitations to this study. The retrospective nature of our study cannot accurately address the complex, real-time clinical judgements and interactions between patient, parent, nurse, and physician postoperatively. There was a small sample size of AR and SS groups used in propensity score matching; however, our sensitivity test confirmed there was no significant differences in pain score or opioid consumption between compared groups. The patient’s place of residence, length of time residing in the United States, cultural background, and socioeconomic status were not collected since they were not available at the time of retrospective data collection, and may impact the patient–provider relationship and expectations. Furthermore, while it has been shown that race and ethnicity can play a role in disparities in pain management, race and ethnicity data were incomplete and hence, this was not included as a covariate in this analysis. Though we strove to study the effect of primary language on opioid consumption and pain scores, it was possible that there were some biases as a result of a patient’s race or ethnicity that impacted our data by underrepresenting moderate to severe pain. Parental satisfaction in each of our study groups was unattainable and unknown. A majority of pain scores reported relied on nonverbal cues from patients, with most scores (87%) being FLACC scores. While verbal cues were not readily used in most patients, it was possible that parental language could have played a part in observer scoring. Finally, an additional unobtainable piece of information was the primary language of the healthcare providers, including the post anesthesia care unit, intensive care unit, and floor nurses, who were interacting with these patients and their families. The potential benefit for enhanced patient–provider interactions between those with concordant primary language could alter the results.

## 5. Conclusions

Parental primary language did not impact pain recognition and pain management in our EA and TEF patient population. Standardization of surgical technique, anesthetic management, pain assessment, and postoperative management may have played a role in our results. Future prospective studies that assess language discordant parent–provider relationships and its impact on perioperative pediatric pain management, and patient or parent satisfaction, are needed.

## Figures and Tables

**Table 1:** Opioid Equivalents and Pain Scores by Language Prior to Propensity Score Matching.

Outcome	English Group (*n* = 537)	Arabic Group (*n* = 45)	Spanish Group (*n* = 28)	*p* Value—English vs. Arabic	*p* Value—English vs. Spanish
Opioid Equivalents per kg					
Intraoperative (*n* = 535, *n* = 45, *n* = 28)	0.77 (0.29, 1.54)	0.92 (0.25, 1.72)	1.38 (0.54, 2.36)	0.771	0.011 *
6 h (*n* = 537, *n* = 45, *n* = 28)	0.15 (0.02, 0.35)	0.17 (0.02, 0.34)	0.23 (0.09, 0.49)	0.881	0.045 *
12 h (*n* = 537, *n* = 45, *n* = 28)	0.16 (0.01, 0.37)	0.20 (0.04, 0.46)	0.29 (0.10, 0.66)	0.543	0.043 *
24 h (*n* = 537, *n* = 45, *n* = 28)	0.27 (0.02, 0.72)	0.36 (0.06, 0.87)	0.38 (0.21, 1.09)	0.197	0.021 *
36 h (*n* = 537, *n* = 45, *n* = 28)	0.21 (0.01, 0.67)	0.39 (0.03, 0.91)	0.42 (0.11, 1.27)	0.109	0.018 *
48 h postop. (*n* = 515, *n* = 43, *n* = 28)	1.15 (0.12, 2.78)	1.52 (0.43, 3.83)	1.68 (0.75, 4.22)	0.184	0.035 *
Pain Scores					
First Pain Score (*n* = 446, *n* = 30, *n* = 21)	0 (0, 4)	0 (0, 0)	0 (0, 5)	0.003 *	0.523
6 h (*n* = 388, *n* = 25, *n* = 18)	1 (0, 3)	0 (0, 0)	0.25 (0, 4.5)	0.001 *	0.761
12 h (*n* = 372, *n* = 28, *n* = 17)	1.5 (0, 3.5)	0 (0, 1)	1 (0, 3.5)	0.002 *	0.99
24 h (*n* = 390, *n* = 25, *n* = 19)	1.5 (0, 3)	0 (0, 2)	1 (0, 3)	0.197	0.808
36 h (*n* = 389, *n* = 26, *n* = 19)	1 (0, 3)	0.5 (0, 2)	0 (0, 2)	0.224	0.16
48 h (*n* = 397, *n* = 26, *n* = 18)	0 (0, 2)	0 (0, 1.5)	0 (0, 2)	0.336	0.493

Data are presented as median (interquartile range). Sample sizes for patients with non-missing pain scores data (n) in each group are indicated. *p* values were calculated using the Wilcoxon rank sum test. * Statistically significant. The bold for *p*-values.

**Table 2:** Patient Characteristics Before and After Propensity Score Matching Between English- and Arabic-Speaking Patients.

Before Propensity Score Matching				
Variable	English Group (*n* = 537)	Arabic Group (*n* = 45)	*p* Value	SMD
Age (years)	0.89 (0.28, 3.05)	1.32 (0.61, 4.11)	0.019 *	0.105
Weight (kg)	7.89 (4.5, 14.1)	8.04 (5.39, 16.2)	0.387	0.016
Sex				
Female	208 (38.7%)	19 (42.2%)	0.645	0.071
Male	329 (61.3%)	26 (57.8%)		
Surgical Procedures—CPT Codes				
31750	37 (6.9%)	2 (4.4%)	0.759	0.105
31760	367 (68.3%)	30 (66.7%)	0.817	0.036
33800	244 (45.4%)	22 (48.9%)	0.655	0.069
43305	16 (3%)	4 (8.9%)	0.060	0.250
43312	91 (17%)	11 (24.4%)	0.204	0.185
43313	127 (23.7%)	18 (40%)	0.015 *	0.354
43314	97 (18.1%)	5 (11.1%)	0.309	0.197
Encounter				
First	419 (78%)	34 (75.6%)	0.702	0.058
Second	119 (22%)	11 (24.4%)		
Developmental Delay	163 (30.4%)	27 (60%)	<0.001 *	0.62
History of Chronic Pain	3 (0.6%)	1 (2.2%)	0.276	0.141
**After Propensity Score Matching (5 English-Speaking Patients to 1 Arabic-Speaking Patient)**				
**Variable**	**English Group (*n* = 195)**	**Arabic Group (*n* = 39)**	***p* Value**	**SMD**
Age (years)	0.96 (0.36, 2.77)	1.32 (0.66, 4.25)	0.050	0.051
Weight (kg)	8.39 (5.3, 13)	8.6 (5.39, 16.5)	0.336	0.059
Sex				
Female	80 (41%)	18 (46.2%)	0.553	0.103
Male	115 (59%)	21 (53.8%)		
Surgical Procedures—CPT Codes				
31750	8 (4.1%)	2 (5.1%)	0.675	0.048
31760	139 (71.3%)	25 (64.1%)	0.371	0.153
33800	93 (47.7%)	18 (46.2%)	0.861	0.031
43305	8 (4.1%)	3 (7.7%)	0.399	0.151
43312	48 (24.6%)	8 (20.5%)	0.684	0.098
43313	64 (32.8%)	14 (35.9%)	0.713	0.064
43314	25 (12.8%)	5 (12.8%)	0.999	0
Encounter				
First	145 (74.4%)	39 (74.4%)	0.999	0
Second	50 (25.6%)	10 (25.6%)		
Developmental Delay	104 (53.3%)	21 (53.9%)	0.999	0.01
History of Chronic Pain	3 (1.5%)	1 (2.6%)	0.520	0.072

Continuous data are presented as median (interquartile range), and categorical data are presented as n (%) within each group on the encounter level. *p* values were calculated using the Wilcoxon rank sum test, the Chi-square test, or Fisher’s exact test, as appropriate. A standardized mean difference value less than 0.2 is considered as reflecting good balance between the two groups. * Statistically significant. The bold for *p*-values. SMD, absolute standardized mean difference.

**Table 3:** Patient Characteristics Before and After Propensity Score Matching Between English- and Spanish-Speaking Patients.

Before Propensity Score Matching				
Variable	English Group (*n* = 537)	Spanish Group (*n* = 28)	*p* Value	SMD
Age (years)	0.89 (0.28, 3.05)	0.70 (0.29, 1.51)	0.477	0.055
Weight (kg)	7.89 (4.5, 14.1)	6.2 (5.26, 10.65)	0.457	0.287
Sex				
Female	208 (38.7%)	13 (46.4%)	0.432	0.155
Male	329 (61.3%)	15 (53.6%)		
Surgical Procedures—CPT Codes				
31750	37 (6.9%)	3 (10.7%)	0.439	0.134
31760	367 (68.3%)	18 (64.3%)	0.679	0.085
33800	244 (45.4%)	14 (50%)	0.637	0.091
43305	16 (3%)	2 (7.1%)	0.223	0.188
43312	91 (17%)	6 (21.4%)	0.605	0.113
43313	127 (23.7%)	6 (21.4%)	0.999	0.053
43314	97 (18.1%)	5 (17.9%)	0.999	0.005
Encounter				
First	419 (78%)	20 (71.4%)	0.484	0.151
Second	119 (22%)	8 (28.6%)		
Developmental Delay	162 (30.2%)	12 (42.9%)	0.156	0.263
History of Chronic Pain	3 (0.6%)	0 (0%)	0.999	0.106
**After Propensity Score Matching (5 English-Speaking Patients to 1 Spanish-Speaking Patient)**				
**Variable**	**English Group (*n* = 135)**	**Spanish Group (*n* = 27)**	***p* Value**	**SMD**
Age (years)	0.69 (0.23, 1.83)	0.69 (0.27, 1.39)	0.821	0.013
Weight (kg)	7.1 (4.4, 11.1)	6.2 (5.3, 10.4)	0.795	0.033
Sex				
Female	50 (37%)	12 (44.4%)	0.47	0.149
Male	85 (63%)	15 (55.6%)		
Surgical Procedures—CPT Codes				
31750	12 (8.9%)	2 (7.4%)	0.999	0.054
31760	85 (63%)	18 (66.7%)	0.828	0.077
33800	66 (48.9%)	14 (51.9%)	0.835	0.059
43305	4 (3%)	1 (3.7%)	0.999	0.041
43312	27 (20%)	6 (22.2%)	0.796	0.054
43313	35 (25.9%)	6 (22.2%)	0.811	0.086
43314	25 (18.5%)	5 (18.5%)	0.999	0
Encounter				
First	96 (71.1%)	19 (70.4%)	0.999	0.016
Second	39 (28.9%)	8 (29.6%)		
Developmental Delay	53 (39.3%)	12 (44.4%)	0.67	0.11
History of Chronic Pain	0 (0%)	0 (0%)	0.999	0

Continuous data are presented as median (interquartile range), and categorical data are presented as n (%) within each group on the encounter level. *p* values were calculated using the Wilcoxon rank sum test, the Chi-square test, or Fisher’s exact test, as appropriate. A standardized mean difference value less than 0.2 is considered as reflecting good balance between the two groups. SMD, absolute standardized mean difference.

**Table 4:** Multivariable Median Regression Analysis of Outcomes Compared by Language Group.

Outcome	Adjusted Difference in Medians—Arabic vs. English (95% CI)	*p* Value	Adjusted Difference in Medians—Spanish vs. English (95% CI)	*p* Value
Opioid Equivalents per kg				
Intraoperative	−0.04 (−0.35, 0.27)	0.793	0.25 (−0.36, 0.86)	0.421
6 h	0.03 (−0.04, 0.09)	0.461	0.03 (−0.08, 0.13)	0.591
12 h	0 (−0.1, 0.1)	0.926	0.09 (−0.18, 0.35)	0.51
24 h	0.01 (−0.11, 0.13)	0.848	0 (−0.16, 0.16)	0.988
36 h	0.01 (−0.11, 0.13)	0.877	0.03 (−0.14, 0.21)	0.71
48 h postop.	−0.01 (−0.54, 0.52)	0.975	0.04 (−0.92, 1.01)	0.929
Pain Scores				
First Pain Score	−0.21 (−0.49, 0.08)	0.153	0.01 (−0.29, 0.31)	0.933
6 h	−0.55 (−1.15, 0.06)	0.077	−0.3 (−1.02, 0.43)	0.425
12 h	−0.97 (−1.61, −0.33)	0.003 *	0.1 (−1.52, 1.71)	0.908
24 h	−1.38 (−2.02, −0.75)	<0.001 *	−0.12 (−1.5, 1.25)	0.859
36 h	−0.45 (−1.32, 0.42)	0.308	−0.69 (−1.4, 0.03)	0.061
48 h	−0.06 (−0.4, 0.28)	0.745	−0.2 (−0.68, 0.28)	0.417

Adjusted differences in medians with 95% confidence intervals and *p* values were calculated using multivariable median regression. Each model was adjusted for age, weight, sex, procedure, encounter number, developmental delay, history of chronic pain, regional use, opioid infusions, surgical duration, ventilation, and ICU admission. * Statistically significant. The bold for *p*-values. CI, confidence interval.

## Data Availability

Data available upon request.

## Appendix A. ICD 9 and 10 Codes Queried for Developmental Delay and Pain Disorders

315.4 development coordination disorder
315.5 mixed development disorder
315.8 other specified delays in development
315.9 unspecified delay in development
313.89 other emotional disturbances of childhood or adolescence
338.29 other chronic pain
338.4 chronic pain syndrome
338.19 other acute pain
780.98 generalized pain
783.42 delayed milestones
783.40 unspecified lack pf normal physiological development
F94.9 Childhood disorder of social functioning, unspecified
R62.0 Delayed milestone in childhood
F81.9 Developmental disorder of scholastic skills, unspecified
F88 Other disorders of psychological development
R62.59/R62.50 Other lack of expected normal physiological development in childhood/Unspecified lack of expected normal in childhood
F82 Specific developmental disorder of motor function
F89 Unspecified disorder of psychological development
G89.21 chronic pain due to trauma
G89.4 chronic pain syndrome
G89.22 chronic post-thoracotomy pain
G89.3 neoplasm related pain (acute) (chronic)
G89.29 other chronic pain
G89.28 other chronic postprocedural pain
R52 Pain, unspecified/generalized chronic body pains
M54.6 Pain in thoracic spine
